# Correction: Variation and selection on codon usage bias across an entire subphylum

**DOI:** 10.1371/journal.pgen.1009824

**Published:** 2021-09-27

**Authors:** Abigail L. LaBella, Dana A. Opulente, Jacob L. Steenwyk, Chris Todd Hittinger, Antonis Rokas

The caption for Fig 5 is incorrect. Please see the correct Fig 5 caption here.

**Fig 5:**
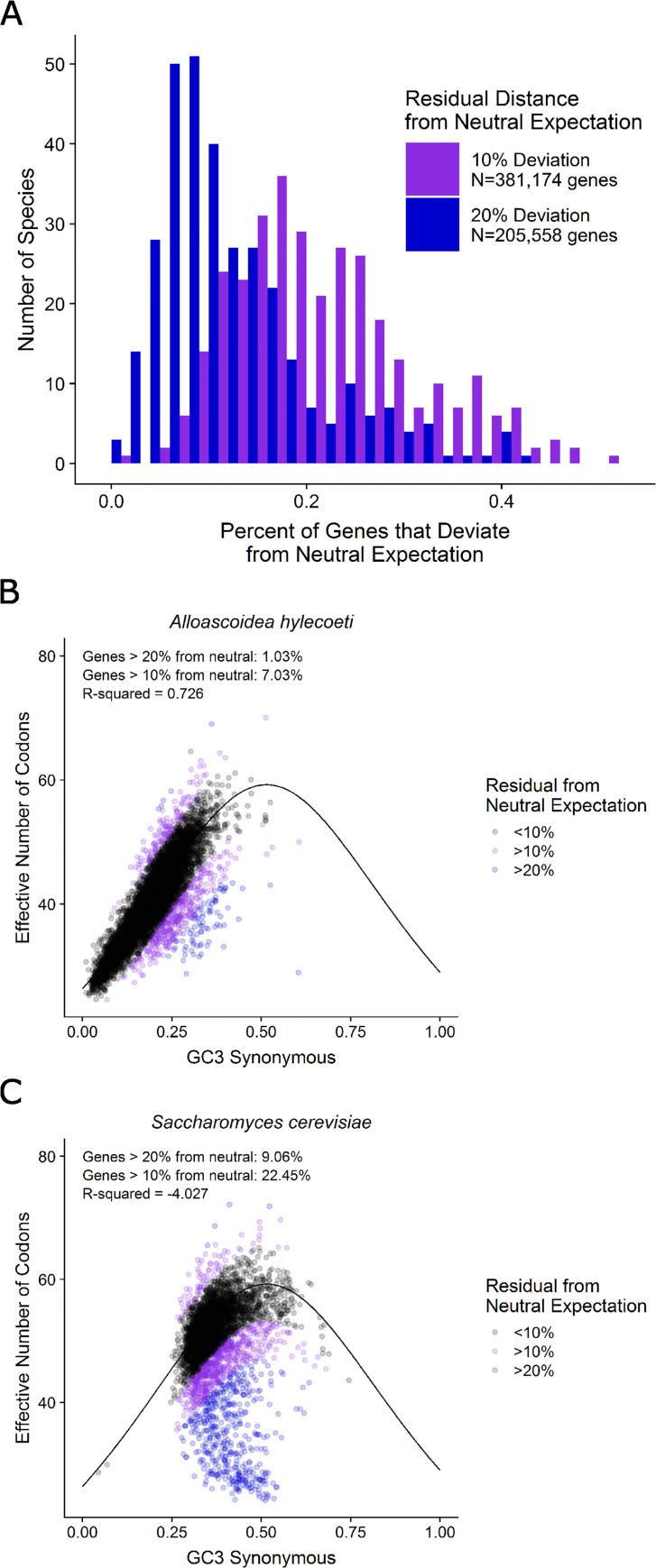
Comparison of the silent third position GC composition of the third codon position (GC3) suggests that individual codons vary in their fit to the neutral expectation (i.e., that codon usage is solely driven by GC mutational bias and genetic drift). Comparison of the silent third position GC composition (GC3s) to the effective number of codons (Nc) across 327 budding yeast species shows that a significant portion of the genes in many species’ genomes deviate substantially from the neutral expectation. A) Distribution of the percentage of genes that deviate more than 10% (purple bars) or 20% (blue bars) from the neutral expectation. Almost half of the genomes have 10% or more of their genes deviate at the 20% threshold (159 / 327), and almost all of the genomes do so at the 10% threshold (309 / 327). B) The genome of the yeast Alloascoidea hylecoeti shows a high correlation between GC3s and Nc (R-squared value = 0.762), in line with neutral expectations. The neutral expectation (i.e., the expectation when the only influence is GC mutational bias and genetic drift) of the effective number of codons for a given GC content of third positions in a genome is indicated by the black line. C) In contrast, the genome of Saccharomyces cerevisiae shows a lack of correlation between GC3s and Nc (R-squared value = -4.027) and does not conform with the neutral expectation.
