# Supplementary figures and images for: Variation and selection on codon usage bias across an entire subphylum
Source: PLoS Genet. 2019 Jul 31;15(7):e1008304. doi: 10.1371/journal.pgen.1008304 (PMC6701816; doi:10.1371/journal.pgen.1008304)

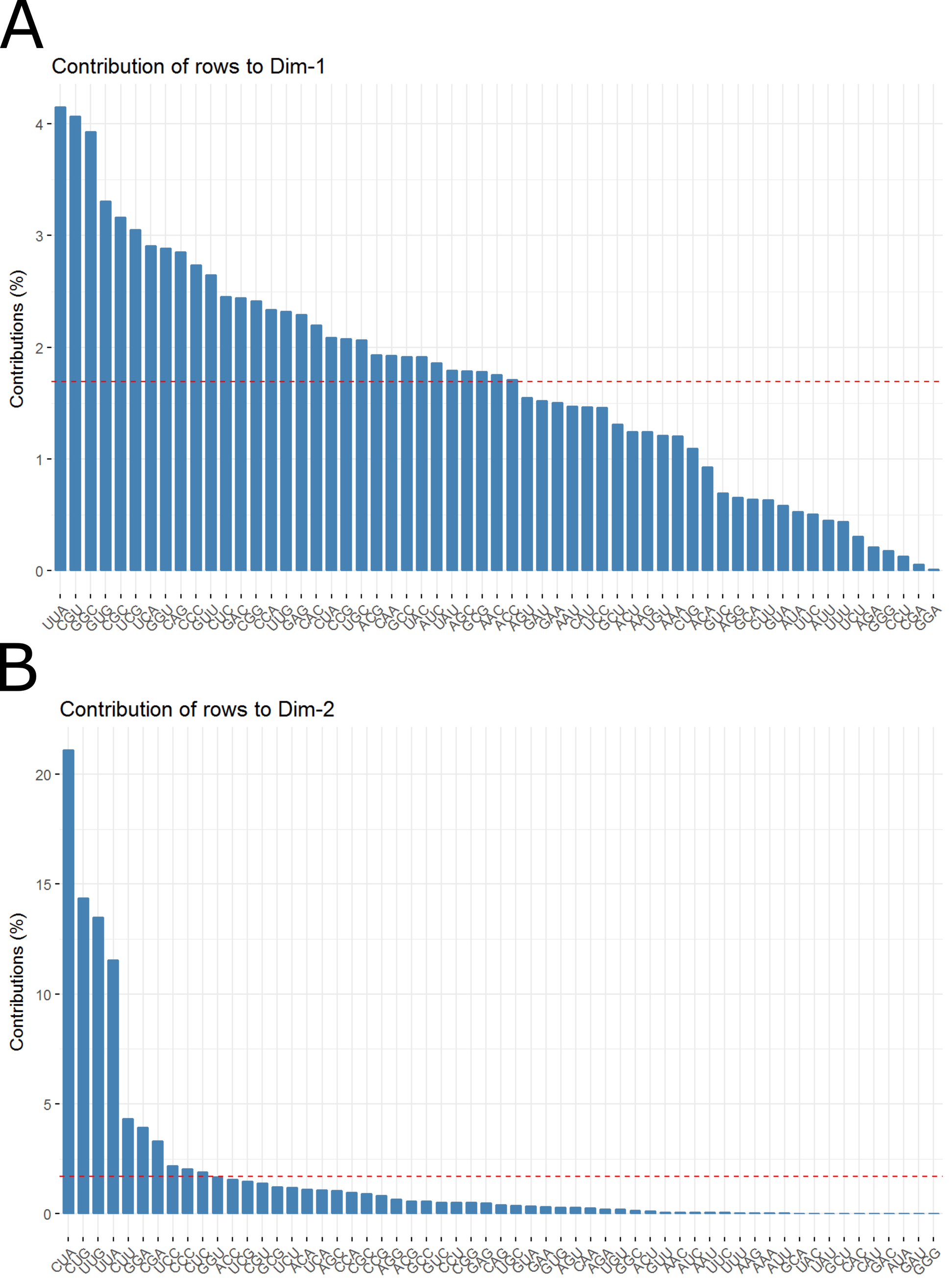

Supplement: S1 Fig — A) The contributions of codons to the first dimension (66.891% of the overall variation) was distributed among multiple codons. Each of the codons made a relatively small contribution to the variation but collectively accounted for most of the differences in RSCU observed between species. B) The contributions of codons to the second dimension (7.093% of the overall variation) was dominated by four codons that contribute more than 10% each to the variation. (TIF) [file pgen.1008304.s001.tif]

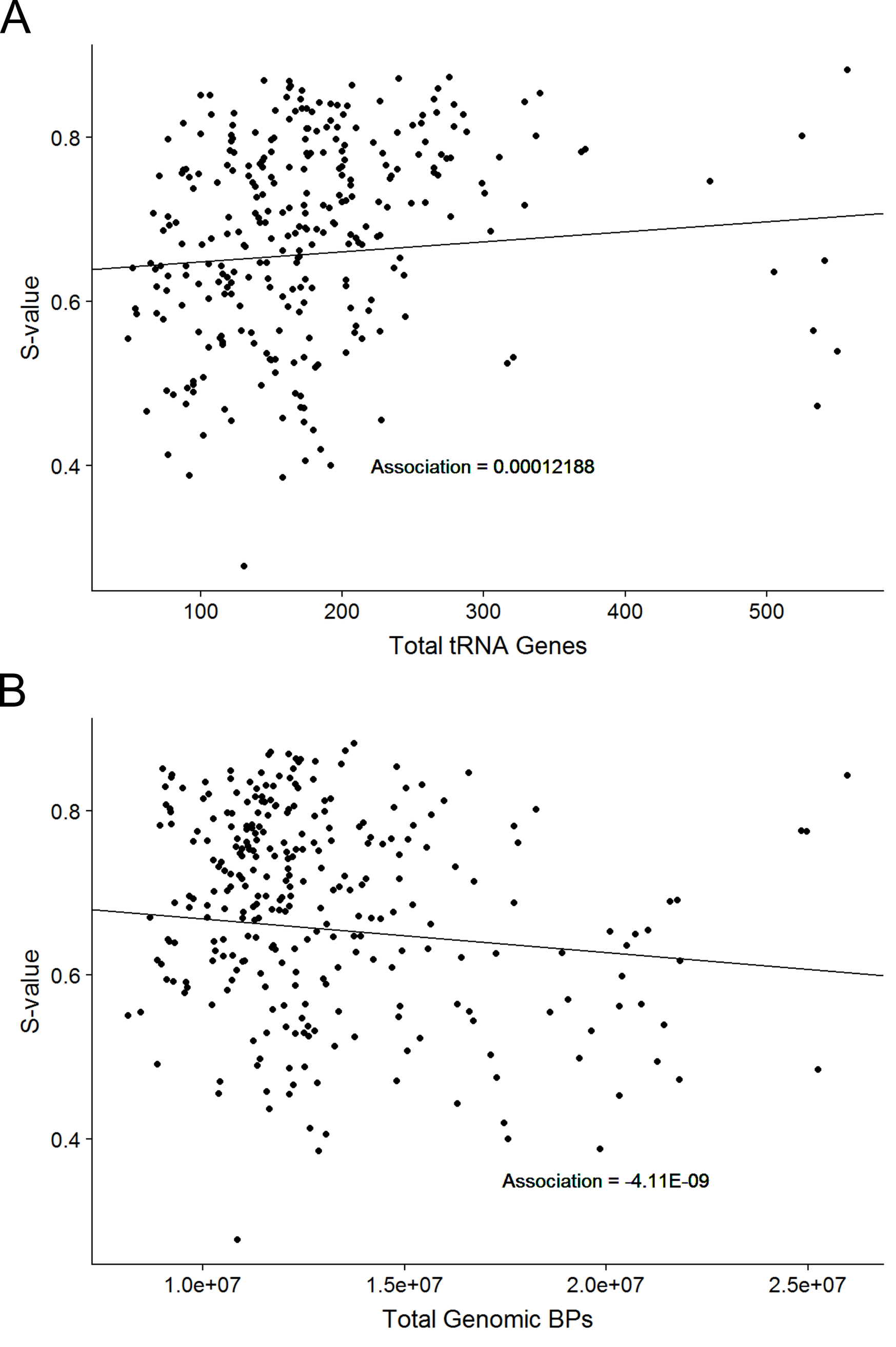

Supplement: S2 Fig — A) The association between number of tRNA genes and S-value (slope = 0.00012) after correction for phylogenetic relatedness was nearly flat, suggesting that total tRNA genes do not linearly reflect the selective pressure on codon bias within a genome. B) The association between genome size (in base pairs) was nearly flat (slope ~ 0), suggesting that, after phylogenetic correction, there is no relationship between genome size and the selective pressure on codon bias within a genome. (TIF) [file pgen.1008304.s002.tif]

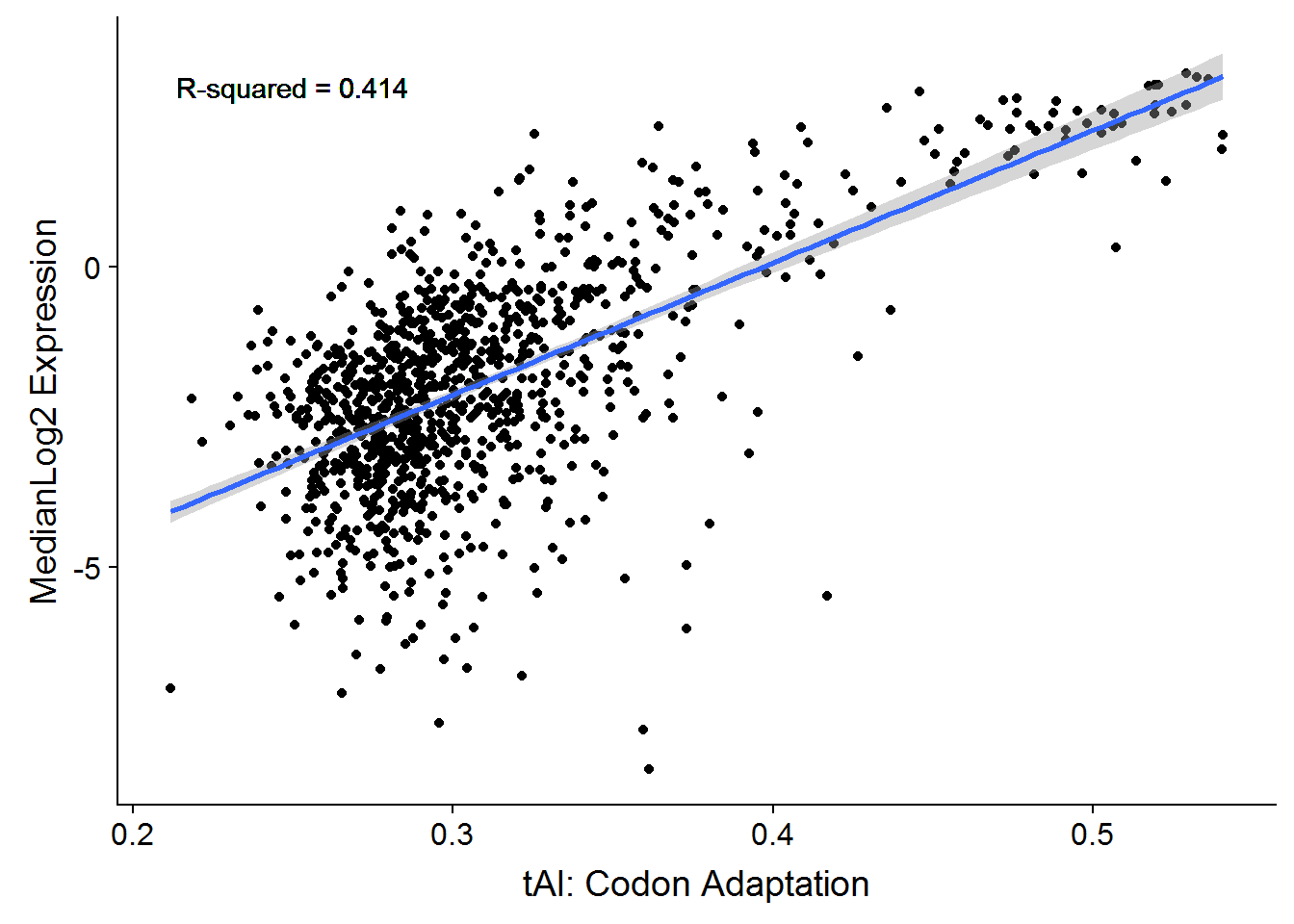

Supplement: S3 Fig — For each gene in the Saccharomyces mikatae genome we measure codon adaptation to the tRNA pool (tAI). This is positively correlated with expression at steady-state. (TIF) [file pgen.1008304.s003.tif]
